# Phase I study of hypofractionated intensity modulated radiation therapy with concurrent and adjuvant temozolomide in patients with glioblastoma multiforme

**DOI:** 10.1186/1748-717X-8-38

**Published:** 2013-02-20

**Authors:** Noha Jastaniyah, Albert Murtha, Nadeem Pervez, Duc Le, Wilson Roa, Samir Patel, Marc Mackenzie, Dorcas Fulton, Colin Field, Sunita Ghosh, Gino Fallone, Bassam Abdulkarim

**Affiliations:** 1 Division of Radiation Oncology, Cross Cancer Institute and University of Alberta, 11560, University Avenue, Edmonton, AB, T6G 1Z2, Canada; 2 Medical Physics, Cross Cancer Institute and University of Alberta, 11560, University Avenue, Edmonton, AB, T6G 1Z2, Canada; 3 Division of Statistics and Epidemiology, Cross Cancer Institute and University of Alberta, 11560, University Avenue, Edmonton, AB, T6G 1Z2, Canada; 4 Department of Oncology, Division of Radiation Oncology, Montreal General Hospital, McGill University, 1650 Avenue Cedar, Montréal, QC, H3G 1A4, Canada

**Keywords:** GBM, Concurrent RT and TMZ, Hypofractionation, IMRT, Phase I study

## Abstract

**Purpose:**

To determine the safety and efficacy of hypofractionated intensity modulated radiation therapy (Hypo-IMRT) using helical tomotherapy (HT) with concurrent low dose temozolomide (TMZ) followed by adjuvant TMZ in patients with glioblastoma multiforme (GBM).

**Methods and materials:**

Adult patients with GBM and KPS > 70 were prospectively enrolled between 2005 and 2007 in this phase I study. The Fibonacci dose escalation protocol was implemented to establish a safe radiation fractionation regimen. The protocol defined radiation therapy (RT) dose level I as 54.4 Gy in 20 fractions over 4 weeks and dose level II as 60 Gy in 22 fractions over 4.5 weeks. Concurrent TMZ followed by adjuvant TMZ was given according to the Stupp regimen. The primary endpoints were feasibility and safety of Hypo-IMRT with concurrent TMZ. Secondary endpoints included progression free survival (PFS), pattern of failure, overall survival (OS) and incidence of pseudoprogression. The latter was defined as clinical or radiological suggestion of tumour progression within three months of radiation completion followed by spontaneous recovery of the patient.

**Results:**

A total of 25 patients were prospectively enrolled with a median follow-up of 12.4 months. The median age at diagnosis was 53 years. Based on recursive partitioning analysis (RPA) criteria, 16%, 52% and 32% of the patients were RPA class III, class IV and class V, respectively. All patients completed concurrent RT and TMZ, and 19 patients (76.0%) received adjuvant TMZ. The median OS was 15.67 months (95% CI 11.56 - 20.04) and the median PFS was 6.7 months (95% CI 4.0 – 14.0). The median time between surgery and start of RT was 44 days (range of 28 to 77 days). Delaying radiation therapy by more than 6 weeks after surgery was an independent prognostic factor associated with a worse OS (4.0 vs. 16.1 months, *P* = 0.027). All recurrences occurred within 2 cm of the original gross tumour volume (GTV). No cases of pseudoprogression were identified in our cohort of patients. Three patients tolerated dose level I with no dose limiting toxicity and hence the remainder of the patients were treated with dose level II according to the dose escalation protocol. Grade 3–4 hematological toxicity was limited to two patients and one patient developed Grade 4 Pneumocystis jiroveci pneumonia.

**Conclusion:**

Hypo-IMRT using HT given with concurrent TMZ is feasible and safe. The median OS and PFS are comparable to those observed with conventional fractionation. Hypofractionated radiation therapy offers the advantage of a shorter treatment period which is imperative in this group of patients with limited life expectancy.

## Introduction

GBM, a WHO grade IV glioma, is the most common and most malignant primary central nervous system tumour. Currently, the standard of care for these patients is safe, maximal surgical resection, if possible, followed by postoperative RT with concomitant TMZ followed by six cycles of adjuvant TMZ as established by the 22981/26981–EORTC/NCIC phase III trial [1]. The median survival with this multimodality treatment is approximately 14 months [1].

The standard radiation dose is 60 Gy in 1.8-2.0 Gy per fraction given over 6 weeks. Most dose escalation studies were done before the era of intensity modulated radiation therapy (IMRT). Two main studies have examined the use of IMRT in delivering hypofractionated postoperative radiation therapy alone [2,3]. The use of IMRT is considered to allow selective delivery of high dose per fraction to the target volume while maintaining the dose to surrounding normal tissues below certain threshold doses [2]. Moreover, hypofractionation confers the benefits of abbreviating the RT course in patients with a limited life expectancy, reducing costs, and possibly increasing malignant cell killing and decreasing accelerated repopulation [2,3]. Recently, Panet-Raymond et al. [4] retrospectively studied the feasibility of delivering Hypo-IMRT with concurrent and adjuvant TMZ on a cohort of 35 patients. The median survival was found to be comparable to that after conventional fractionation and the regimen was tolerable with no undue toxicity. The use of helical tomotherapy (HT) can provide different geometric flexibility and modulation techniques over standard IMRT. Such superior control of dose distribution could allow for better dose uniformity within the target and/ or sparing of organs at risk [5].

The optimal hypofractionation regimen using IMRT is yet to be determined. In the study by Floyd et al. [2], a dose of 50 Gy at 5 Gy per fraction was given to the enhancing primary tumour, residual disease or surgical cavity with a simultaneous dose of 30 Gy at 3 Gy per fraction to the surrounding oedema. A high rate of cerebral necrosis was noted using this regimen which resulted in earlier termination of patient enrolment. Sultamen et al. [3] prescribed 60 Gy in 20 fractions to the GTV and 40 Gy in 20 fractions to the PTV in their study. In this study, one patient developed blindness 9 months after the completion of radiation treatment but according to the author, it was not felt to be secondary to RT as the dose to the optic chiasm in this patient was 40 Gy with a dose per fraction of 2 Gy.

Our study is one of the few studies that examine the efficacy and safety of delivering Hypo-IMRT using HT with concomitant and adjuvant TMZ in patients with GBM. In this phase I study, a dose escalation protocol using two dose levels, was also used to determine a safe radiation fractionation regimen. Given the toxicity reported in the above mentioned studies, we elected to maintain the dose per fraction below 3 Gy for both dose levels, [2,3]. The delivery of Hypo-IMRT with concomitant and adjuvant TMZ was found to be safe and feasible. The PFS and OS were comparable to those reported with conventional fractionation regimen.

## Materials and methods

### Patients

Adult patients with a histologically confirmed diagnosis of GBM were enrolled between 2005 and 2007. Eligibility criteria included age ≥ 18, KPS ≥ 70, no prior RT to the head or neck area, no prior use of chemotherapy or radio-sensitizer drugs, and normal hematologic, hepatic and renal function. Ethics approval was obtained from Alberta Cancer Ethics Review Board and all patients signed a study-specific consent form. All patients were initially evaluated with a complete history and a detailed neurological evaluation and underwent a computerized tomography (CT) scan and/ or magnetic resonance imaging (MRI) preoperatively. Extent of surgical resection was recorded based on operative report and postoperative imaging.

### Radiation therapy

All patients were treated with HT using a 6 MV linear accelerator. Patients were immobilized with a thermoplastic mask of the head, neck and upper shoulders for simulation and treatment. Patients underwent CT simulation where all irradiated tissues were imaged using 0.3 cm thick slices. An MRI in the treatment position with the same immobilization device was also obtained and fused with the CT images. The GTV was defined as areas of contrast enhancement on T1-weighted images on registered MRI images. The clinical target volume (CTV) included the GTV with a non-uniform 1 cm margin, excluding areas extending beyond the dura, calvarium or other anatomical boundaries. The planning target volume (PTV) was taken as the CTV plus a 0.5 cm margin. A dose escalation protocol was implemented. This mandated treating at least 3 patients using dose level I which was defined as 54.4 Gy in 20 fractions over 4 weeks. The biologically effective dose (BED) of which was calculated to be 62.2 Gy at 2 Gy per fraction based on the linear-quadratic model with the α/β ratio assumed to be 10 Gy for the tumour. The absence of any dose limiting toxicity, defined as an acute Grade 4 neurological radiation-induced toxicity within 30 days of starting radiation, in all of the 3 patients allowed moving into dose level II which was defined as 60 Gy in 22 fractions over 4.5 weeks. The BED of dose level II was calculated to be 68.6 at 2 Gy per fraction based on the same principle. The presence of a single case with a dose limiting toxicity required treating an additional three patients with dose level I and an additional dose limiting toxicity mandated dose reduction for all further patients to 51.4 Gy in19 fractions. The dose was prescribed to at least 95% of the PTV and was delivered in a single phase. Organs at risk (OAR) including the optic nerves, optic chiasm, optic apparatus (right and left optic nerves and optic chiasm), brainstem, spinal cord and right and left eyes were contoured in each slice. Treatment plans were generated using the Hi-Art II System (TomoTherapy Inc., Madison, WI). The jaw width, pitch, and modulation factor used for our study were 2.5, 0.430, and 3 cm, respectively. The dose calculation grid resolution was 2 mm. The average number of iterations for a particular plan was approximately 20. The planning objective was to deliver the specified dose to at least 95% of the PTV while minimizing the dose to the defined OAR. For critical structures, the maximum importance was given to the optic chiasm followed by the brainstem, optic nerves, and spinal cord (if the cord was considered to be in the vicinity of the tumor). The dose constraints for the aforementioned organs were a maximum dose of 50 Gy for brainstem, 40 Gy for spinal cord, 45 Gy for the optic chiasm and optic nerves, and 10 Gy for the orbits. Dose-volume histograms were generated for all critical structures to ensure that the dose delivered was within accepted tolerance levels. A megavoltage CT scan was obtained prior to each fraction for verification of treatment position. A more than 5 mm displacement of the isocenter mandated repositioning of the patient. At our institution, for dose quality assurance (DQA), a cheese phantom is used to measure film and ionisation chamber dosimetry for each RT plan prior to commencing treatment. Criteria for a satisfactory treatment delivery include a point dose measurement, taken in a high dose and low gradient region within 2% of the calculated dose and gamma index with a distance to agreement goal of 3 mm and a percent dose difference goal of 5%. These criteria have been verified at our institution and the details of the DQA process have been previously published [6].

### Chemotherapy

Patients were given TMZ at dose of 75 mg/m2/ day concomitant with RT, beginning the day of the first fraction of radiation, including weekends and holidays and ending on the day of the last radiation fraction. This was followed by up to 6 cycles of adjuvant TMZ at a dose of 150–200 mg/ m2 daily for 5 days every 28 days, as per the EORTC/NCIC protocol [1]. All of the patients received Trimethoprim-Sulfamethoxazole for prophylaxis against Pneumocystis jiroveci pneumonia.

### Patient assessment and outcome

In this phase I study, patients were assessed weekly during RT with clinical examination, Karnofsky performance status, complete blood counts, renal function tests and liver enzymes. After completion of RT, patients were followed-up every three months with clinical evaluation and an MRI. Blood chemistry was obtained at the 3- and 6-months visits and as needed thereafter. The common terminology criteria for adverse events (CTC-AE) were used for grading acute and late side effects. When disease progression was suspected on clinical basis, an MRI was done for confirmation. Response to treatment was classified according to the RECIST criteria. The primary endpoints were feasibility and safety of Hypo-IMRT with concurrent TMZ. The feasibility of the study was assessed by determining the ability to enroll our predetermjined sample size, practicality of planning and delivering Hypo-IMRT using HT, and the ability to achieve the specified planning goals with the use of HT. Secondary endpoints included PFS, pattern of failure, OS and incidence of pseudoprogression. PFS was defined from the day of surgery till the documentation of disease progression clinically and/ or radiologically. OS was measured from registration till death. Pseudoprogression was defined as clinical or radiological suggestion of tumor progression within three months of radiation completion followed by spontaneous recovery of the patient [7].

### Statistical analysis

Descriptive analysis was conducted for all study variables. Mean, standard deviation, median (range) were reported for continuous variables, frequency and percentages were reported for the categorical variables. PFS and OS were analyzed using the Kaplan-Meier method. A univariate analysis using the Cox proportional regression analysis was used to examine the effect of multiple prognostic factors on PFS and OS. These factors included KPS, recursive partitioning analysis (RPA) class [8], type of surgery performed (biopsy vs. subtotal or gross total resection), time interval between the surgery and start of RT and number of adjuvant Temozolomide cycles. Factors identified to have p value ≤ 0.05 on univariate analysis were then analyzed using the multivariate Cox model. All reported p values are two-sided and differences were considered statistically significant when the p value was <0.05. The SAS program (Version 9.1, SAS Institute Inc., Cary, NC, USA) was used for the statistical analysis.

## Results

### Patient characteristics

Between April 2005 and July 2007, 25 patients with histologically proven GBM were prospectively treated with Hypo-IMRT using HT, with concurrent low dose TMZ chemotherapy followed by adjuvant TMZ according to the above-described protocol. The median follow-up of patients included in the analysis was 12.4 months (range, 1.7 to 38.5 months). The patients’ baseline characteristics are summarized in Table 1. The median age at diagnosis was 53.1 years (range, 22.6 - 73.4 years). Patients were classified according to the RTOG RPA criteria. Almost half of the patients (52%) were RPA class IV and the remaining was either class III (16%) or V (32%). Twelve, three and nine patients underwent subtotal resection, gross total resection and biopsy only, respectively.

**Table 1:** Patients characteristics

**Variables**	**No**	**%**
Age at diagnosis		
Median	53 years	
Range	22 - 73 years	
**Gender**		
Male	14	56
Female	11	44
**RPA class**		
III	4	16
IV	13	52
V	8	32
**KPS**		
70	9	36
80	7	28
90	6	24
100	3	12
**Lobe involved**		
Cortical	23	92
Central (pineal, cerebellum)	2	8
**Type of surgery**		
Gross total resection	3	12
Subtotal resection	12	52
Biopsy only	9	36
**Concomitant TMZ**		
Yes	25	100
No	0	0
**Adjuvant TMZ**		
Yes	19	76
No	6	24
Patients who completed >2 cycles	12	48

Abbreviations: *RPA*: recursive partitioning analysis; *KPS*: Karnofsky perfomans status; *TMZ*: Temozolamide.

### Treatment goals and treatment delivery

All of the patients completed RT with concurrent TMZ with no treatment delay or interruption. The median time for starting RT from the date of surgery was 43 days (range, 28–77 days). Three patients were treated with dose level I (54.4 Gy in 20 fractions over 4 weeks) and none of them developed any of the defined dose-limiting toxicity. This allowed proceeding to dose level II to which the remainder of the patients was treated as per the aforementioned dose escalation protocol. The treatment goals are as described in Table 2.

**Table 2:** Protocol treatment goals

**Volume**	**Goal 1**	**Goal 2**	**Goal 3**
PTV	60 Gy to ≥ 95% of the PTV	≤ 20% of the PTV to > 66 Gy	≤ 1% of the PTV to < 54 Gy
Optic Chiasm	≤ 5% to > 47.25 Gy	≤ 33% to 40.5 Gy	
Optic nerves	≤ 5% to > 47.25 Gy	≤ 33% to 40.5 Gy	
Brainstem	≤ 5% to > 52.5 Gy	≤ 33% to 45 Gy	
Spinal cord	≤ 5% to > 42 Gy	≤ 33% to 36 Gy	
Eyes	≤ 5% to > 10.5 Gy	≤ 33% to 9 Gy	

Abbreviations: *PTV*: planning target volume.

The dose–volume analysis for the PTV and OARs is showed in Table 3. The PTV volume ranged from 102.8 to 427.7 cm^3^ (mean 264.3 ± 84.7 cm3). The mean dose to the PTV was 60.8 Gy (SD ± 2.7; range 43.6 - 65.6). The maximum dose received by the optic chiasm, optic nerves and brain stem was 40.5 Gy ± 7.6, 34.6 Gy ± 11.5, and 50.7 Gy ± 10.3, respectively. The dose to the spinal cord was only reported when pertinent and the maximum was 3.1 Gy ± 2.4. Seventy six percent of the treated cohort received adjuvant TMZ but only 48% of the patients completed 3 or more cycles. Of the patients who failed to complete 6 cycles of adjuvant TMZ, 14 of them were due to disease progression. Two patients declined further therapy and treatment was discontinued due to treatment related side effects in three patients, two of them had persistent thrombocytopenia.

**Table 3:** Dose–volume analysis for planning target volume and organs at risk

**Volume**	**Parameter**	**Mean ± SD**
**PTV**	Mean dose	60.8 ± 2.7 Gy
	Minimum dose	43.6 ± 9.4 Gy
	Maximum dose	65.6 ± 4.2 Gy
	V95%	97.9± 2.5%
	Homogeneity index (D5/D95)	1.1 ± 0 (Range 1.0 - 1.2)
**Optic chiasm**	Mean dose	30.4 ± 7.2 Gy
	Maximum dose	40.5 ± 7.6 Gy
	V40	13.9 ± 15.9%
	V45	0.8% ± 1.9%
	V47.25	0%
	V50	0%
**Left optic nerve**	Mean dose	18.3 ± 8.0 Gy
	Maximum dose	29.5 ± 11.1 Gy
	V40	3.6 ± 9.1%
	V47.25	0.1 ± 0.3%
	V50	0
**Right optic nerve**	Mean dose	23.4 ± 10.3 Gy
	Maximum dose	34.6 ± 11.5 Gy
	V40	13.6 ± 21.8%
	V47.25	0%
	V50	0%
**Brainstem**	Mean dose	22.8 ± 10.2 Gy
	Maximum dose	50.7 ± 10.3 Gy
	V40	19.8 ± 19%
	V50	4.5 ± 7.4%
	V52.5	1.3 ± 3%
	V55	0.4 ± 1%
**Spinal cord**	Mean dose	1.2 ± 0.8 Gy
	Maximum dose	3.1 ± 2.4 Gy
	V40	0%
	V42	0%
**Left eye**	Mean dose	5.2 ± 2.8 Gy
	Maximum dose	11.1 ± 4.1 Gy
	V10.5	5 ± 17.7%
**Right eye**	Mean dose	5.7 ± 3.7 Gy
	Maximum dose	13.3 ± 6.4 Gy
	V10.5	5.6 ± 17%

### Treatment toxicity

Concurrent RT and TMZ related acute side effects are reported in Table 4. One patient developed Pneumocystis jiroveci pneumonia infection requiring admission to the intensive care unit (ICU) for ventilatory support. Grade 3 and/or 4 myelosuppressive toxicity developed in two patients precluding the ability to give adjuvant TMZ. The remainder of the patients tolerated the treatment with mild or no acute toxicity.

**Table 4:** Acute toxicity associated with concurrent RT/ TMZ

		**Grade**		
**Toxicity**	**1**	**2**	**3**	**4**
**Gastrointestinal**	1	6	0	0
**Hematological**				
Thrombocytopenia	0	0	1	1
Neutropenia	0	2	1	0
Anemia	1	0	1	0
**Infection**	0	2	0	1

Abbreviations: *RT*: radiation therapy; *TMZ*: temozolamide.

### Prognostic factors and outcome

**Figure 1:**
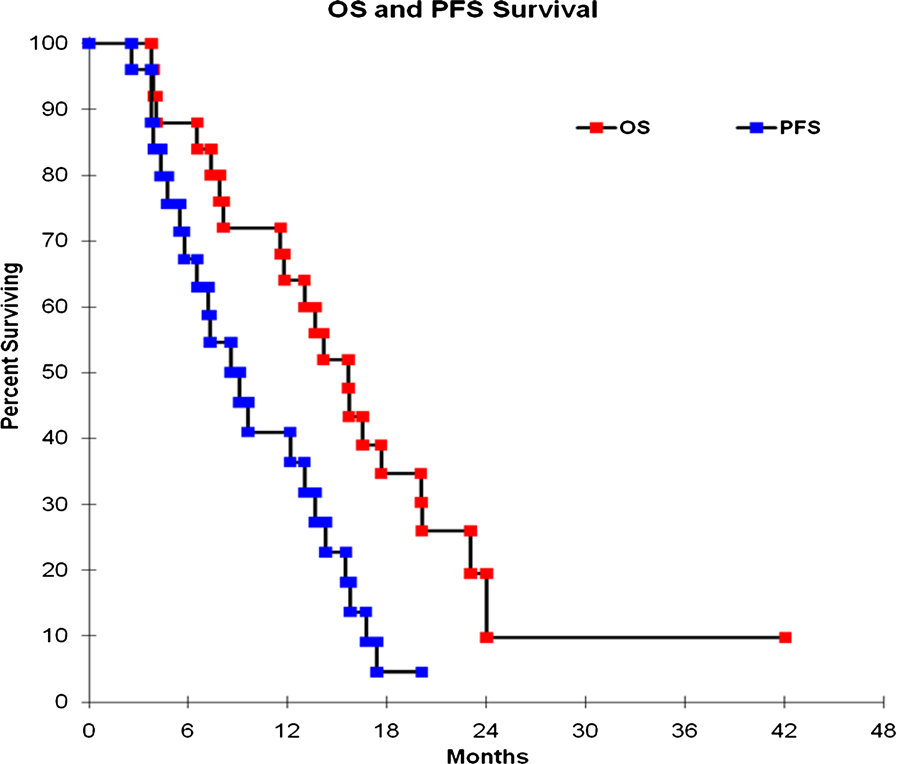
Kaplan-Meier curve for overall survival and progression free survival.

At a median follow-up of 12.4 months, the median OS was 15.67 months (95% CI 11.56 - 20.04) and the median PFS was 6.7 months (95% CI 4.0 – 14.0) (Figure 1). PFS at 6 months was 60% (95% CI 40 – 78) and PFS at 12 months was 38% (95% CI 20 – 58). All the cases of disease recurrence were either local or marginal i.e. within 2 cm of the tumour bed. Nine patients progressed within three months of the completion of RT. We did closely investigate these cases to avoid overlooking cases of pseudoprogression. However, none was identified in our cohort of patients. We investigated the potential association between several prognostic factors and increased risk of GBM progression. A univariate analysis was performed on the following factors: KPS (≥80 vs. < 80), RPA class (class III vs. class IV and V), type of surgery performed (biopsy vs. subtotal or gross total resection), time interval between the surgery and start of RT (≥ 6 weeks vs. < 6 weeks), and number of adjuvant Temozolomide cycles (administration of <3 vs. ≥3 cycles). On multivariate analysis, the administration of 3 or more cycles of adjuvant TMZ (*versus* <3 cycles) was associated with better OS (HR 0.83 [95% CI 0.69-0.99]; *P*-value 0.042) and better PFS (HR 0.79 [95% CI 0.65-0.96]; *P*-value 0.020). In addition, ≥ 6 weeks elapsed time between surgery and the start of radiation therapy (*versus* < 6 weeks) was predictive of worse OS (HR 2.94 [95% CI 1.06-8.18]; *P*-value 0.039). Furthermore, higher KPS ≥ 80 (*versus* < 80) was associated with improved PFS (HR 0.29 [95% CI 0.09-1.00]; *P*-value 0.049). These results are summarized in Tables 5 and 6.

**Table 5:** Univariate and multivariate analysis of prognostic factors for PFS

	**Univariate**			**Multivariate**		
**Variable**	**HR**	**95% CI**	***P***	**HR**	**95% CI**	***P***
**KPS**						
≥ 80 vs. 70	0.4	0.16-0.99	0.048	0.29	0.09-1.00	0.049
**RPA class**						
Class III vs. Class IV and V	0.34	0.10-1.11	0.075			NS
**Adjuvant TMZ**						
≥3 cycles vs. < 3 cycles	0.84	0.72-0.982	0.028	0.79	0.65-0.96	0.02
**Extent of resection**						
GTR vs. biopsy or STR	0.54	0.15-1.91	0.339			NS
**Time interval between the date of surgery and start of RT**						
≥ 6 weeks vs. < 6 weeks	1.08	0.45-2.59	0.867			NS

Abbreviations: *PFS*: progression free survival; *HR*: hazard ration; *CI*: confidence interval; *KPS*: Karnofsky performance status; *RPA*: recursive partitioning analysis; *TMZ*: Temozolomide; *GTR*: gross total resection; *STR*: subtotal resection; *RT*: radiation therapy.

**Table 6:** Univariate and multivariate analysis of prognostic factors for OS

	**Univariate**			**Multivariate**		
**Variable**	**HR**	**95% CI**	***P***	**HR**	**95% CI**	***P***
**KPS**						
≥ 80 vs. 70	1.32	0.50-3.46	0.573			NS
**RPA class**						
Class III vs. Class IV and V	2.51	0.56-11.20	0.228			NS
**Adjuvant TMZ**						
≥3 cycles vs. < 3 cycles	0.79	0.64-0.97	0.024	0.83	0.69-0.99	0.042
**Extent of resection**						
GTR vs. biopsy or STR	1.99	0.45-8.86	0.368			NS
**Time interval between the date of surgery and start of RT**						
≥ 6 weeks vs. < 6 weeks	3.18	1.20-8.40	0.02	2.94	1.06-8.18	0.039

Abbreviations: *OS*: overall survival; *HR*: hazard ration; *CI*: confidence interval; *KPS*: Karnofsky performance status; *RPA*: recursive partitioning analysis; *TMZ*: Temozolomide; *GTR*: gross total resection; *STR*: subtotal resection; *RT*: radiation therapy.

## Discussion

Hypo-IMRT using HT is an approach that combines high-precision RT delivery and a hypofractionated regimen. Patients with GBM have a dismal prognosis and a limited life span. The period of best performance is even shorter as clinical deterioration is associated with profound morbidity. Thus, achieving similar clinical outcome while abbreviating treatment course can be of great clinical significance. An additional advantage to hypofractionation is the potential of improving tumour control. Our study is the first to prospectively examine the use of Hypo-IMRT while administering concurrent and adjuvant TMZ for newly diagnosed GBM patients. The efficacy and safety of this regimen were demonstrated by this phase I study. Our data are consistent with the results reported by Stupp et al. [1] with a median OS of 15.67 months a median PFS of 6.7 months. The study’s inability to show improved outcome, over standard fractionation, could be due to our reluctance to proceed to higher dose levels or merely a reflection of the small sample size tested.

The risk of neurological side effects, particularly radionecrosis of the brain is considered to be the main deterrent of using a hypofractionated scheme. The threshold fraction size above which this risk is clinically significant is difficult to determine. One reason is the considerable variation in the fractionation regimens used [9] and the major difference in the prognostic characteristics of the patients in whom the regimen was studied. In addition, most of the dose escalation literature is based on conventional methods of radiation delivery. The use of IMRT, HT in particular, is thought to physically allow dose escalation while keeping reasonable dose constraints to at risk normal tissue. Two main studies have prospectively investigated the use of IMRT to deliver a hypofractionated radiation therapy to patients with GBM and each used a different regimen [2,3]. Floyd et al. [2], used a dose of 50 Gy at 5 Gy per fraction given to the enhancing primary tumour, residual disease or surgical cavity with a simultaneous dose of 30 Gy at 3 Gy per fraction to the surrounding oedema. Twenty percent of the patients evaluated for late toxicity experienced Grade 4 cerebral necrosis. The latter study by Sultamen et al. [3] prescribed 60 Gy in 20 fractions to the GTV and 40 Gy in 20 fractions to the PTV in their study. One patient developed blindness 9 months after the completion of radiation treatment but the aetiology of the visual loss was not felt to be attributable to RT. There is a wide range between the fractions sizes used in these studies. Currently, the wide recognition of concurrent and adjuvant TMZ as the standard of care for GBM, diminishes somewhat the applicability of these previous studies to current treatment considerations. The concern of an increased risk of cerebral radionecrosis with the addition of chemotherapy is a valid one based on previous reports [7]. Panet-Raymond et al. [4] did a retrospective analysis on 35 patients who were treated with post-operative Hypo-IMRT and TMZ according to the Stupp protocol. In this study a total dose of 60 Gy in 20 daily 3-Gy fractions was delivered to the GTV while ensuring that the 95–100% isodose line covered the GTV, and the 65–70% line encompassed the PTV. The median overall survival was 14.4 months, and the median disease-free survival was 7.7 months, both of which were comparable to those reported by the EORTC/NCIC trial [1]. No late toxicity was seen in that cohort of patients but considering their method of prescribing the dose, the PTV could have received a dose per fraction as low as 2 Gy which is not different from conventional fractionation. In our study, we used a dose escalation protocol to establish a safe and tolerable fractionation regimen and no late toxicity was reported with either dose levels. Of interest, a recently published phase I trial of Hypo-IMRT with TMZ used a dose escalation protocol [10]. The study included 16 patients and examined four dose levels. A total dose of 60 Gy in 3 Gy/fraction, 4 Gy/fraction, 5 Gy/fraction, and 6 Gy/fraction, was prescribed in dose levels 1, 2, 3 and 4, respectively. One patient who was treated at level 2 with 60 Gy in 4 Gy/fraction developed Grade 4 visual loss at 7 months following RT. Three patients developed pathologically confirmed extensive necrosis. One was treated at level 1, one at level 2 and the third at level 4. Therefore, most of the up-to-date hypofractionation studies suggest that fraction sizes larger than 3 Gy could be highly associated with detrimental late effects.

With regard to acute toxicity, two patients in our study developed Grade 3–4 myelosuppression which is expected given the toxicity profile reported in the EORTC/NCIC trial [1]. One patient developed severe Pneumocystis jiroveci pneumonia infection requiring ICU admission. This has been previously reported in patients with brain tumours [11] and because of which prophylaxis against this opportunistic infection was mandatory for all patients receiving TMZ in the EORTC/NCIC trial. All of our patients completed concurrent chemoradiation but only 76% of the patients received adjuvant TMZ. The median number of adjuvant TMZ cycles was 2 and in the EORTC/NCIC trial, the median number was 3. However, due to the small sample size of our study, it is difficult to conclude whether the intensified radiation dose could have reduced the patients’ tolerance to adjuvant TMZ. Two patients had persistent thrombocytopenia precluding the use of adjuvant TMZ. The impact of thrombocytopenia was examined in a retrospective analysis of 52 consecutive patients with newly diagnosed high-grade gliomas treated with the Stupp protocol [12]. The rate of Grade 3–4 thrombocytopenia was found to be 19% with a significant risk of prolonged, possibly irreversible, low platelet count. On multivariate analysis, we found that the administration of 3 or more cycles of adjuvant TMZ was associated with better OS and PFS. Our current guidelines suggest 6 cycles of adjuvant TMZ as per EORTC/NCIC trial [1]. However, the optimal duration of this adjuvant therapy, beyond six months, is currently being prospectively investigated.

Despite the use of conformal RT, disease recurrence occurs in the treatment volume in the majority of patients [13]. The rate of recurrence outside the tumour bed is low and mostly occurs with or after recurrence of the original disease [14]. Brandes et al. [15] has recently reported the pattern of failure in 95 patients with newly diagnosed GBM treated with radiotherapy plus concomitant and adjuvant TMZ. A shift in the pattern of failure from local or marginal locations to distant locations was observed. Recurrences outside the treatment field were observed in 20% of the patients. Furthermore, there was a significant higher survival rate in patients with recurrence outside the RT fields (median survival of 26.1 months vs. 17.3 months). This change in the recurrence pattern was not mirrored in our study which could be due to the small sample size of our cohort. The same study by Brandes et al. [15] also reported strong correlation between the recurrence pattern and the MGMT methylation status. The rate of infield recurrence correlated significantly with MGMT methylation status. The rate was higher in patients with unmethylated MGMT versus those with methylated MGMT (85% vs. 57.9%). This factor could not be assessed in our study as MGMT methylation was unfortunately not tested in our patients.

The incidence of pseudoprogression has been variably reported in the literature. Brandes et al. [16] reported the incidence of pseudoprogression among 103 patients treated with TMZ concurrent with and adjuvant to RT. Out of 50 patients who developed radiological progression as assessed by MRI done 4 weeks after treatment completion, 32 (64%) were identified to have pseudoprogression. On the other hand, the data published by Sanghera et al. [17] on a series of 104 patients, only 7 patients (32%) were found to have pseudoprogression out of 22 classified to have early disease progression as indicated by an MRI 8 weeks post-RT. During the 3 month period after the concurrent treatment, radiation-induced brain injury could be associated with an increase of non-enhancing and enhancing tumour component on MRI. In our study, unless otherwise clinically indicated, an MRI was first routinely performed after three months of the end of RT. Accordingly, only nine cases were classified as early progression and all of which were true disease progression. This could reflect the fact that neuroradiological imaging was avoided during the early period following RT during which radiation induced CNS injury, manifested as an increase in contrast enhancement and/or non-enhancing tumour components, is at its peak.

The RPA class is one of the most significant prognostic factors that determine survival in patients with GBM. This has also been shown to hold true in the era of concurrent RT and TMZ (18). Unexpectedly, the RPA in our cohort of patients did not correlate with survival. The most likely explanation is that only patients with a KPS ≥70 were included in our study. This resulted in clustering of the majority of patients within one RPA class and due to our small sample size; the effect of the RPA class could not be statistically appreciated. Blumenthal et al. reported a large analysis on the effect of the time to initiate RT following surgery for GBM patients (19). Approximately, three thousand patients, from the Radiation Therapy Oncology Group (RTOG) database, were examined. The median survival was found to be significantly greater in patients in whom RT was started more than 4 weeks from surgery. All of the patients started RT within 6 weeks as mandated by all the study protocols according to these patients were treated. The authors of the study concluded that short delays in initiating RT, not exceeding 6 weeks, may not significantly affect survival but cautioned that the reported observation could reflect physician’s tendency to expedite treatment in patients with worse outlooks resulting in apparently worse outcome when RT was started earlier. In our study, delays greater than 6 weeks in initiating RT adversely affected outcome. The influence of delaying RT could have been potentiated by the presence of considerable proportion (36%) of patients who only had a biopsy for a tumour notorious for its short doubling time.

In summary, the results of this phase I dose-escalation study have shown that Hypo-IMRT, using HT, with concurrent TMZ is safe and feasible. Our data are consistent with results reported EORTC/NCIC trial, with a median OS of 15.67 months a median PFS of 6.7 months. The Hypo-IMRT regimen is shorter than standard RT schedules. Abbreviating the RT regimen can be clinically meaningful considering the life expectancy of patients with GBM. It is hoped that these advanced technologies can provide better quality of life, more convenient treatment options, and optimal utilization of the resources by health care providers. Our findings warrant further validation of the results by conducting a phase II randomized controlled trial comparing our treatment regimen to conventional fractionation. Careful progression through higher dose levels, in the setting of a clinical trial seems possible.

## Competing interest

The authors indicated no potential conflicts of interest.

## Authors’ contribution

NJ carried out data collection and writing article; NP protocol writing; DL data collection; AL, WR, SP, and DF patient recruitment; MM, CF and GF carried out medical physics data and treatment planning; SG carried out statistical analysis and BA protocol design, patient recruitment, data analysis and writing article. All authors read and approved the final manuscript.
